# Porto-mesenteric vein thrombosis after laparoscopic sleeve gastrectomy. A case report

**DOI:** 10.1016/j.ijscr.2021.01.086

**Published:** 2021-01-26

**Authors:** Ozan Şen, Simay Kara, Ahmet Gökhan Türkçapar

**Affiliations:** a Türkçapar Bariatrics, Obesity Center, Dikilitaş Mah. Ayazmadere cad, Yeşil Çimen sok no: 9, Beşiktaş, 34394, İstanbul, Turkey; b Nişantaşı University, Department of Health Sciences, Maslak mah. Söğütözü sok. no: 20, Maslak 1453, Sarıyer, 34398, İstanbul, Turkey; c Acibadem Fulya Hospital, Radiology Department, Hakkı yeten cad. Yeşilçimen sok. No: 23, 34349, İstanbul, Turkey

**Keywords:** Bariatric surgery, Laparoscopic sleeve gastrectomy, Complication, Porto-Mesenteric vein trombosis

## Abstract

**İntroduction and importance:**

Porta-mesenteric vein thrombosis (PMVT) is a rare but fatal complication in patients who are undergoing bariatric surgery. In this report, we present a rare case of a PMVT after laparoscopic sleeve gastrectomy (LSG).

**Case presentation:**

A 52-year-old male patient with a body mass index of 42 kg/m^2^ was admitted to our clinic for morbid obesity. Standart LSG was performed with 5 trocar technique. 15 days after LSG, the patient admitted to the emergency department with complaints of abdominal pain, nausea and vomiting. The patient was dehydrated. His C-reactive protein level was 138 mg/L. Abdominal computerized tomography with contrast was performed and showed thickening of a part of small bowel wall in 10 cm length. Also, major trombosis were detected in the superior mesenteric vein branches and portal vein. The patient was hospitalized and 2 × 10,000 IU/1.0 mL high dosage low moleculer weight heparin (LMWH) therapy was initiated. The patient’s clinical signs recovered rapidly following treatment.

**Clinical discussion:**

İn LSG, if the gastroepiploic venous arcus, which runs along the greater curvatura, and has a direct connection to the portal circulation is damaged, a local thrombus may form and move towards the portal system over time. Dehydration is another significant predisposing factor for PMVT. Some patients may develop life-threatening intestinal ischemia. Abdominal tomography with contrast plays a major role in diagnosis.

**Conclusion:**

PMVT should be considered as a serious complication after LSG in patients with abdominal pain. With early diagnosis and anticoagulant therapy, patients’s clinical symptoms may improve quicly.

## Highlights

• Porta-mesenteric vein thrombosis (PMVT) is a serious complication that maybe seen after sleeve gastrectomy.
• Abdominal pain is the most significant symptom of PMVT.
• Some patients may develop life-threatening intestinal ischemic symptoms.
• Early diagnosis and anticoagulant therapy are very important.

## 1 Introduction

Bariatric surgery has been shown to be the most effective treatment for the management of morbid obesity [1]. Among the bariatric surgical methods, laparoscopic sleeve gastrectomy (LSG) has become the most common method worldwide in recent years [2]. Despite its popularity, there may be some complications post LSG. The most serious complications after LSG are leak (1%–3.9%), bleeding (<5%), and stenosis (2%–5%) [3,4].

Mesenteric or portal vein thrombosis is a rare but fatal complication in patients who are undergoing bariatric surgery. Its incidence has been reported as 0.3% in all bariatric surgical procedures [5]. The first symptom is generaly abdominal pain for this complication in most patients. This pain can be felt in the back or under the left scapula. Nausea and vomiting also may accompany to the abdominal pain [5]. Abdominal computerized tomography with constrast has a significant role in diagnosis. Emergency abdominal expolaration may be required in some patients with signs of intestinal ischemia or peritonitis [5]. Early diagnosis and treatment are crucial. Proper hydration, bowel rest and anticoagulant therapy may help regress most of these symptoms. In this report, we present a rare case of PMVT after LSG.

This case report has been reported in line with SCARE criteria [15].

## 2 Case presentation

A 52-year-old male patient with a body mass index of 42 kg/m^2^ was admitted to our clinic for morbid obesity. He had a previous history of insulin resistance and hypertension and was on medication for these conditions. The patient was screened by detailed laboratory tests with respect to hematologic and metabolic parameters and vitamin deficiency (iron, Ferritin, B12, Folic acid, Vitamine D). There was no vitamin deficiency but hiperlipidemia was found. Gastroscopy and abdominal ultrasound were also performed at preoperative evaluation. There was no patology in the stomach. He had grade 2 hepathosteatosis. He was evaluated by a multidisciplinary team like all our patients. (Dietitian, psychiatrist, cardiologist, pulmonologist, endocrinologist and anesthetist). After preoperative evaluation LSG was planned. The patient was informed about the operation and this study in detail, and written consents were obtained.

### 2.1 Venos thromboembolism prophylaxis

As a routine treatment, this patient received low molecular weight heparin (LMWH), 40 mg enoxaparin sodium, which was repeated every 24 h for 10 days. Early postoperative mobilization, the use of pneumatic compression sucks during surgery and at hospitalization period were also applied as our routine standart procedure.

### 2.2 Surgery

The 12 mm optic trocar (Endopeth Xcel®) was entered into the abdomen under direct vision from the left supcostal area and insufflated with 12 mmHg CO_2_. LSG was performed with 5 trocars. Lesser suck was opened and greator curvature was disected using harmonic scalpel. The stomach was completely released. LSG was completed using Endo GIA TM60 mm Tri-stapler (4 purple and 1brown cartridge) starting from 2 cm distance to the pylorus using 36 French bougie. Then the entire stapler line was oversewed with 3.0 V-Loc TM. At the last stage, gastric sleeve was fixed to the peripancreatic tissue at 2 points below the incisura to prevent twist. The opereration lasted for 90 min.

## 3 Results

The patient had no problem in the postoperative period and was discharged on the third postoperative day. Venous thromboembolism prophylaxis was continued for 7 more days with a daily injection of 40 mg enoxoparin sodium.

**Fig. 1:**
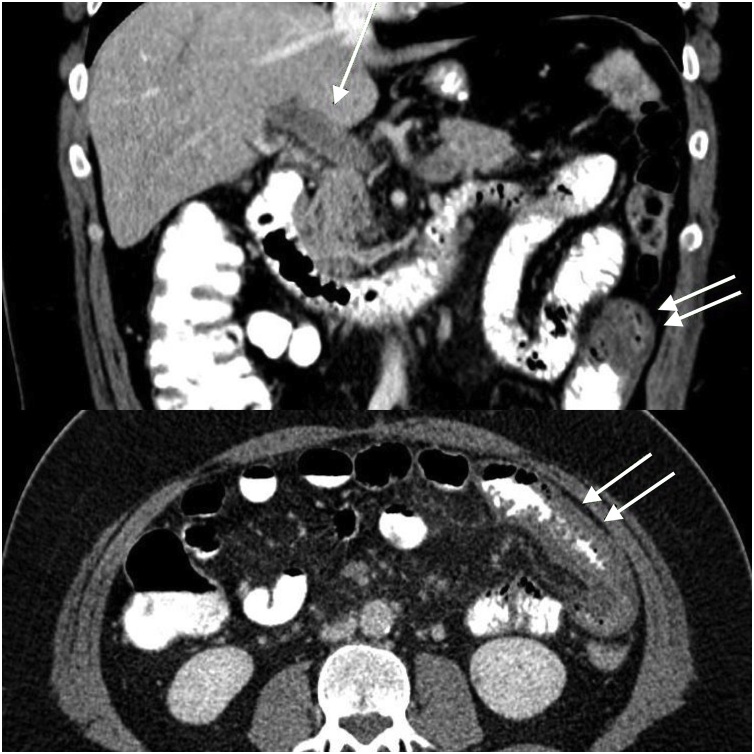
Coronal and axial abdominal computerized tomography section. Single arrow: Thrombus in portal vein, Double arrow: small bowel wall thickening area.

**Fig. 2:**
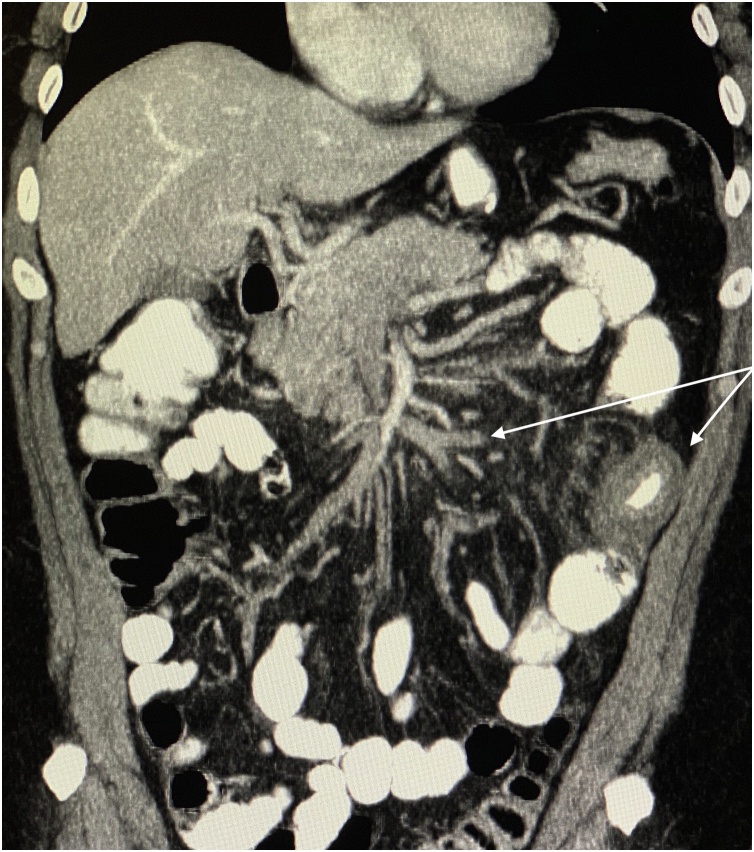
Coronal abdominal computerized tomography section. Long arrow: Thrombus in superior mesenteric vein branchs, Short arrow: İscemic intestinal segment.

15 days after LSG, the patient admitted to the emergency department with complaints of abdominal pain, nausea and vomiting. The patient notified that abdominal pain was perpetual, intensifying especially after eating. Blumberg sign in the left paramedian area of the abdomen was found in physical examination. The patient was dehydrated. His C-reactive protein level was 138 mg/L. (references range 0–5 mg/L). Intravenous hydration was started. Abdominal CT with oral and intravenous contrast was performed. The result of the CT showed thickening of a part of the small bowel wall in 10 cm length. Also, symptoms of mesenteric fatty tissue inflammation were detected. Also, major trombosis were detected in the superior mesenteric vein branches and portal vein (Figs. 1 and 2). The patient was hospitalized. Oral nutrition was stopped. 2 × 10000 IU/1.0 mL low moleculer weight heparin (LMWH) therapy was initiated. The patient’s clinical signs recovered rapidly following treatment and he was discharged after five days with anticoagulant therapy. As a result of hematology consultation, predisposing factors such as Factor V Leiden mutation, Protein C and Protein S resistance that will cause thrombosis were not detected. Oral anticoagulant therapy was planned for 6 months. The patient was monitored for possible portal hypertension that may develop in the future.

## 4 Discussion

The development of PMVT is a rare but fatal complication in patients after LSG. Some basic factors may be determinant for the development of PMVT. Some of these factors are; prothrombinogenic factors (Factor V Leiden mutation, Protein C, S resistance, thrombophilia, using oral contraceptive), elevated intraabdominal pressure in laparoscopic surgery due to CO_2_ insufflation, patient position and long operation time. In addition, the risk of thromboembolism is higher in obese people due to metabolic syndrome and comorbid diseases [6].

It has been reported that incidence of PMVT is 0,3% after the LSG, more frequently than other bariatric procedures [5,7]. There may be some possible reason for the more frequent occurrence of this complication after LSG. In LSG operation, short gastric vessels along the greater curvature are ligated by energy devices (Harmonic scalpel or Ligasure) that is used for releasing the stomach. Meanwhile, the gastroepiploic venous arcus, which runs along the greater curvatura and has a direct connection to the portal circulation may be damaged. A local thrombus may form and move towards the portal system over time [7]. We pay extra attention when cutting short gastric vessels to avoid damaging the gastroepiploic arc in our LSG technique. For this reason, we cut short gastric vessels very close to the stomach wall. When the images of surgery retrospectively were examined in this patient, it was determined that the gastroepiploic arch was not directly damaged. On the other hand, some patients may be dehydrated because of insufficient liquid intake after LSG. Dehydration is a significant predisposing factor for venous thromboembolism [5,8]. A study conducted on 250000 patients who underwent bariatric surgery reported that dehydration was the main reason in admitting to an emergency department [9]. In some clinics, patients are discharged the day after LSG, while we provide 3 days of hospital stay as standard for all our patients. In our practice, patients are discharged after they are able to take enough fluid orally. Nevertheless, patients may not pay much attention to the liquid intake after discharge. It has been determined that liquid intake was not sufficient after discharge in this patient. Moreover, smoking is also a major risk factor for the development of PMVT [5]. Our patient has been an active smoker for 15 years.

Abdominal pain is the most significant symptom of PMVT. Also, nausea and vomiting may be seen in some patients. Some patients may develop life-threatening intestinal ischemia, necrosis, peritonitis, and abdominal sepsis, while the symptoms of the disease are mild in others [5,10,11]. Diagnostic laparotomy and intestinal resection may be required in patients with signs of peritonitis. Abdominal CT with oral and iv contrast has a major role in diagnosis. Its sensitivity is over 90% [5,[10], [11], [12]].

Early diagnosis and treatment are very important in patients with the development of PMVT after bariatric surgery. High-dose of LMWH or unfractionated heparin theraphy should be initiated immediately after diagnosis [5,13]. Sufficient hydration and bowel rest are other parts of the treatment. Clinical symptoms recover with early diagnosis and anticoagulant therapy in most patients. It has been reported that in cases with complete thrombosis, portal vein catheterization with trombolitic therapy application were succesful [5]. In delayed cases, portal hypertension may develop in the future [14]. After starting anticoagulant therapy in our patient, the clinical symptoms improved rapidly. Also hematology evaluation revealed no genetic mutation that would cause thrombosis. Oral anticoagulant therapy was planned for 6-months, and the patient was monitored closely.

## 5 Conclusion

PMVT is a rare but serious complication that maybe seen after LSG. PMVT should be considered after LSG in patients with abdominal pain. Early diagnosis and treatment are very important. Dehydration is one of the most important predisposing factors for this complication. Precautions should be taken to prevent dehydration especially in the early period after LSG.

## Sources of funding

No sources of funding.

## Ethical approval

The study is exempt from ethical approval in our institution.

## Consent

Written informed consent was obtained from the patient for publication of this case report and accompanying images. A copy of the written consent is available for review by the Editor-in-Chief of this journal on request.

## Author's contribution

Each author have participated sufficiently in the work to take public responsibility for appropriate portions of the content. All authors met all of the following criteria:

- Substantial contributions to the conception or design of the work; or the acquisition, analysis, or interpretation of data for the work; OS, SK and AGT
- Drafting the work or revising it critically for important intellectual content; OS
- Final approval of the version to be published; OS, AGT
- Agreement to be accountable for all aspects of the work in ensuring that questions related to the accuracy or integrity of any part of the work are appropriately investigated and resolved.

AT and OS operated the patient.

OS wrote the first draft of the manuscript.

OS and AT wrote the final draft of the manuscript.

OS and SK made the corrections in English. All authors have read and approved the final report.

## Registration of research studies

Not Applicable.

## Guarantor

Ozan Şen, MD.

## Provenance and peer review

Not commissioned, externally peer-reviewed.

## Declaration of Competing Interest

The authors report no declarations of interest.

## References

[1] Sjöström L. (2013). Review of the key results from the Swedish Obese Subjects (SOS) trial-a prospective controlled intervention study of bariatric surgery. J. Intern. Med..

[2] (2018). American Society for Metabolic and Bariatric Surgery estimation of metabolic and bariatric procedures performed in the United States in 2016. Surg. Obes. Relat. Dis..

[3] Lalor P.F., Tucker O.N., Szomstein S. (2008). Complications after laparoscopic sleeve gastrectomy. Surg. Obes. Relat. Dis..

[4] Abd Ellatif M., Abbas A., El Nakeeb A. (2017). Management options for twisted gastric tube after laparoscopic sleeve gastrectomy. Obes. Surg..

[5] Goitein D., Matter I., Raziel A., Keidar A., Hazzan D., Rimon U. (2013). Portomesenteric thrombosis following laparoscopic bariatric surgery: incidence, patterns of clinical presentation, and etiology in a bariatric patient population. JAMA Surg..

[6] Morange P.-E., Alessi M.-C. (2013). Thrombosis in central obesity and metabolic syndrome: mechanisms and epidemiology. Thromb. Haemost..

[7] Rocha A.T., de Vasconcellos A.G., da Luz Neto E.R. (2006). Risk of venous thromboembolism and efficacy of thromboprophylaxis in hospitalized obese medical patients and in obese patients undergoing bariatric surgery. Obes. Surg..

[8] Salinas J., Barros D., Salgado N. (2014). Portomesenteric vein thrombosis after laparoscopic sleeve gastrectomy. Surg. Endosc..

[9] Ivanics Tommy, Nasser Hassan, Leonard-Murali Shravan, Genaw Jeffrey (2019). Dehydration risk factors and impact after bariatric surgery: an analysis using a national database. Surg. Obes. Relat. Dis..

[10] Swartz D.E., Felix E.L. (2004). Acute mesenteric venous thrombosis following laparoscopic roux-en-Y gastric bypass. JSLS.

[11] Singh P., Sharma M., Gandhi K. (2010). Acute mesenteric vein thrombosis after laparoscopic gastric sleeve surgery for morbid obesity. Surg. Obes. Relat. Dis..

[12] Morasch M.D., Ebaugh J.L., Chiou A.C., Matsumura J.S., Pearce W.H., Yao J.S. (2001). Mesenteric venous thrombosis: a changing clinical entity. J. Vasc. Surg..

[13] Joh J.H., Kim D.I. (2005). Mesenteric and portal vein thrombosis: treated with early initiation of anticoagulation. Eur. J. Vasc. Endovasc. Surg..

[14] Plessier A., Darwish-Murad S., Hernandez-Guerra M. (2010). Acute portal vein thrombosis unrelated to cirrhosis: a prospective multicenter follow-up study. Hepatology.

[15] Agha R.A., Franchi T., Sohrabi C., Mathew G., for the SCARE Group (2020). The SCARE 2020 Guideline: Updating Consensus Surgical CAse REport (SCARE) Guidelines. Int. J. Surg..

